# External quality assessment of HIV-1 DNA quantification assays used in the clinical setting in Italy

**DOI:** 10.1038/s41598-022-07196-2

**Published:** 2022-02-28

**Authors:** Ilaria Vicenti, Filippo Dragoni, Alessia Giannini, Anna Casabianca, Francesca Lombardi, Laura Di Sante, Ombretta Turriziani, Sara Racca, Stefania Paolucci, Alessia Lai, Isabella Bon, Isabella Abbate, Gabriella Rozera, Simone Belmonti, Rossana Scutari, Claudia Alteri, Francesco Saladini, Maurizio Zazzi, Italian HIV DNA Network, Chiara Orlandi, Mauro Magnani, Simona Di Giambenedetto, Roberta Longo, Stefano Menzo, Daniele Di Carlo, Laura Mazzuti, Anna Ardemagni, Massimo Clementi, Fausto Baldanti, Federica Giardina, Annalisa Bergna, Claudia Balotta, Alessia Bertoldi, Maria Rosaria Capobianchi, Francesca Ceccherini-Silberstein, Maria Antonello, Carlo Federico Perno, Massimo Andreoni

**Affiliations:** 1 Department of Medical Biotechnologies, University of Siena, Siena, Italy; 2 Department of Biomolecular Sciences, University of Urbino “Carlo Bo”, Urbino, PU Italy; 3 Fondazione Policlinico Universitario A. Gemelli IRCCS, UOC Malattie Infettive, Rome, Italy; 4 Virology Unit, Department of Biomedical Sciences and Public Health, Polytechnic University of Marche, Ancona, Italy; 5 Department of Molecular Medicine, Sapienza University of Rome, Rome, Italy; 6 Laboratory of Microbiology and Virology, IRCCS San Raffaele Scientific Institute, Milan, Italy; 7 Molecular Virology Unit, Microbiology and Virology Department, Fondazione IRCCS Policlinico San Matteo, Pavia, Italy; 8 Department of Biomedical and Clinical Sciences Luigi Sacco, University of Milan, Milan, Italy; 9 UO Microbiologia, IRCSS Azienda Ospedaliero Universitaria di Bologna, Policlinico S. Orsola, Bologna, Italy; 10 Virology and Biosafety Laboratories Unit, National Institute for Infectious Diseases IRCCS L. Spallanzani, Rome, Italy; 11 Dipartimento di Sicurezza e Bioetica, Sezione Malattie Infettive, Università Cattolica del Sacro Cuore, Rome, Italy; 12 Department of Experimental Medicine, University of Rome “Tor Vergata”, Rome, Italy; 13 Department of Oncology and Hemato-Oncology, University of Milan, Milan, Italy; 14 Multimodal Medicine Research Area, Bambino Gesù Children’s Hospital, IRCCS, Rome, Italy; 15 Infectious Diseases Clinic, University Hospital “Tor Vergata”, Rome, Italy

**Keywords:** Retrovirus, Viral reservoirs, Diagnostic markers, DNA

## Abstract

Total cell-associated HIV-1 DNA is a surrogate marker of the HIV-1 reservoir, however, certified systems for its quantification are not available. The Italian HIV DNA Network was launched to validate HIV-1 DNA quantification methods in use at University and Hospital labs. A quality control panel including HIV-1 DNA standards, reconstructed blood samples (RBSs) and DNA from different HIV-1 subtypes was blindly tested by 12 participating labs by quantitative real-time PCR (n = 6), droplet digital PCR (n = 3) or both (n = 3). The median 95% hit rate was 4.6 (3.7–5.5) copies per test and linearity in the tested range was excellent (R^2^ = 1.000 [1.000–1.000]). The median values obtained across labs were 3,370 (2,287–4,245), 445 (299–498), 59 (40–81) and 7 (6–11) HIV-1 DNA copies, for the 3,584, 448, 56 and 7-copy standards, respectively. With RBSs, measured values were within twofold with respect to the median in two thirds of cases. HIV-1 subtypes were missed (CRF01_AE by 3 labs) or underestimated by > 1 log (subtypes A, C, D, F by one lab; CRF01_AE by one lab; CRF02_AG by one lab). The overall performance was excellent with HIV-1 DNA standards, however detection of different HIV-1 subtypes must be improved.

## Introduction

Antiretroviral therapy achieves prolonged control of HIV-1 replication in the vast majority of patients^1^. However, eliminating HIV-1 infection remains an elusive goal due to indefinite persistence of the viral reservoir, characterized by latently infected cells carrying HIV-1 proviral DNA in their host genome^2^. Quantifying the HIV-1 reservoir in patients under successful treatment, as defined by undetectable HIV-1 RNA in plasma, is of great interest because a small sized reservoir would theoretically be suitable for different treatment strategies. On one hand, in patients with a limited HIV-1 reservoir it should be safer to reduce drug pressure with the aim of decreasing treatment toxicity and cost. On the other hand, such patients are likely to be the ideal candidates for pilot HIV-1 eradication studies through strategies targeting the latent HIV-1 reservoir^3^. Thus, reliable and practical markers are needed to analyze the viral reservoir^4^.

Indeed, several systems have been proposed to quantify the viral reservoir^5^. Measuring virus outgrowth following stimulation of patient blood cells in vitro is considered the gold standard to quantify latent but replication competent virus^6^. However, there is no methodological consensus and the assays described differ in one or more features, including the patient cell population and the uninfected cells co-cultured, the approach used for reversing HIV-1 latency and the markers measured to quantify the induced virus^7–9^. In general, such methods are complex, time-consuming and difficult to standardize. In addition, they tend to underestimate the viral reservoir because not all the replication competent virus population can be effectively induced in vitro^10^. While viral outgrowth assays remain very valuable in investigating the nature, dynamics and pathogenesis of the HIV-1 reservoir, lower complexity methods are needed to integrate a measure of the latent HIV-1 reservoir into routine patient management.

Molecular assays such as the quantification of total cell-associated HIV-1 DNA can fulfill these requirements since they obviate the need for cell cultivation, biosafety level containment and high-level, specific expertise^11^. However, total HIV-1 DNA clearly overestimates the viral reservoir since it includes not only replication competent proviruses but also defective and more labile, unintegrated forms^10,12^. Nonetheless, extrachromosomal forms can contribute to HIV-1 pathogenesis^13^ and total HIV-1 DNA load seems to correlate well with the frequency of cells containing replication-competent virus^14,15^. In addition, although discriminating between integrated and unintegrated HIV-1 DNA can add useful information in select studies, assays specifically targeting integrated HIV-1 DNA are complex to set up and require extensive replicate testing which makes the system not amenable to routine use^14,16^. Most importantly, the clinical role of total HIV-1 DNA is supported by sparse but relevant studies. A meta-analysis of six studies in untreated patients^17^ indicated that total HIV-1 DNA is a stronger predictor of AIDS and of all-cause mortality compared to plasma HIV-1 RNA load. In addition, baseline total HIV-1 DNA load is predictive of the occurrence and severity of HIV-1 associated neurologic disorders^18^ as well as of levels of T-cell activation^19^. More recently, the role of total HIV-1 DNA has been evaluated in treated patients with suppressed plasma HIV-1 RNA providing two important lines of evidence. First, total HIV-1 DNA is predictive of the time to plasma HIV-1 RNA rebound after treatment interruption, both in patients treated early during primary infection^20,21^ and in patients treated late during chronic infection^22,23^. Second, higher total HIV-1 DNA levels are associated with an increased risk of virological failure following treatment de-escalation, as shown in the MONOI and MONET trials^24,25^, comparing the outcomes of a switch to darunavir/ritonavir (DRV/r) monotherapy *vs*. combination therapy, and in the DOMONO trial evaluating the switch to DTG monotherapy^26^.

The increasing interest for HIV-1 DNA quantification as a rough estimate of the viral reservoir has not yet been accompanied by the development of certified assays to measure this parameter. Few commercial assays have been developed but they have not been certified for in vitro diagnostic use. However, several homebrew HIV-1 DNA quantification protocols have been developed worldwide. Such assays typically undergo different kinds of internal validation but the results obtained from different methods can be hardly compared due to different genomic regions analyzed, different standards used and/or different principles and readouts, such as real time PCR (qPCR) or droplet digital PCR (ddPCR). The Italian HIV DNA Network was launched to investigate the features and performance of the HIV-1 DNA quantification methods in use at Italian University and Hospital labs, including homebrew systems and commercial kits not yet marked for in vitro diagnostic use. Here we report the results obtained from the first external quality assurance program involving 12 Italian labs receiving a comprehensive panel of HIV-1 DNA standards, reconstructed clinical samples and DNA extracts from different HIV-1 subtypes.

## Methods

### Participating centers

The quality control panel was assembled by the coordinating center and sent to the 12 labs participating to the Italian HIV DNA Network, including the coordinator, for blind testing, using the methodology routinely used at each center. The labs were originally asked to participate to the Network based either on the use of commercial assays or on publication of at least one peer-reviewed paper reporting quantification of HIV-1 DNA.

### Quality control material for the external validation

Detailed instructions concerning the manipulation and processing of the quality assurance sample panel were provided to each lab. The number of replicates, dilutions and results required to validate the assay are indicated in Table 1.

**Table 1:** Composition of the external quality assurance sample panel and output requested for the different parameters of performance.

Parameter	Content	Replicates	Output
Sensitivity	Twofold dilution series containing nominal 1.75 to 112 copies of the reference standard	8 for each dilution (intra-run)	Frequency of positive results
Linear range	Tenfold dilution series containing nominal 7 to 70,000 copies of the reference standard	4 for each dilution (intra-run)	HIV-1 copies/test
Intra-run precision	Eightfold dilution series containing nominal 7 to 3,584 copies of the reference standard	4 for each dilution	HIV-1 copies/test
Inter-run precision	Eightfold dilution series containing nominal 7 to 3,584 copies of the reference standard	5 for each dilution	HIV-1 copies/test
Inter-laboratory reproducibility and intra-laboratory precision	5 reconstructed clinical samples estimated to contain 38,315/23,684, 2,189/1,942, 808/693, 160/162 and 0/0 copies per million cells at the coordinating lab by qPCR/ddPCR	3 DNA extractions for each sample, each quantified in duplicate in 3 separate runs (inter-run)	HIV-1 copies/million cells
Detection of different subtypes	7 DNA extracts from MOLT-4/CCR5 cells infected with different HIV-1 subtypes estimated to contain 664/1,068 (A), 469/948 (CRF01_AE), 102,494/233,579 (CRF02_AG), 31,305/36,765 (B), 97,343/121,256 (C), 70,599/83,835 (D), 214,738/297,904 (F) copies per 10^6^ cells at the coordinating lab by qPCR/ddPCR	A duplicate for each sample in intra-run	HIV-1 copies/million cells

To assess the accuracy, sensitivity, precision and linear range on reference standards, the pNL4-3 plasmid (code ARP2006), obtained from the Centre for AIDS Reagents (CFAR) was quantified with respect to an International Standard (code ARP956; CFAR) by qPCR^27^ and ddPCR, using the same primers and probe as for qPCR in a reaction adapted for the QX200™ Droplet Digital™ PCR System (Bio-Rad), and by fluorometric quantification using the Qubit 4 fluorometer (ThermoFisher), based on the mean of the three measurements which differed from one another within 1.5-fold. The plasmid standard was then diluted in 10 ng/µl of human genomic DNA (code G3041; Promega), and frozen until shipment to each lab.

To assess inter-laboratory variability and intra-laboratory precision on clinical whole blood samples, reconstructed blood samples (RBS) were generated at the coordinator lab by diluting U1/HIV-1 lymphoblastoid cells (code ARP139; CFAR), carrying 2 copies of HIV-1 genome per cell, into HIV-1 negative human peripheral blood (code 297CTIPB.1.5; CTI Biotech), provided by the supplier in compliance with all relevant ethical guidelines. Briefly, U1 cells were counted and one million cells were added to 1 ml of HIV-1-negative blood and then serially diluted 1:10, 1:10, 1:3, 1:3. At the coordinating lab, aliquots of the above dilutions series were processed to extract DNA and quantify HIV-1 DNA to confirm that the HIV-1 DNA amount was in a range suitable for the purpose of the study. The same HIV-1 negative human peripheral blood used as diluent was included in the quality assurance panel to assess the specificity of the assays. Since these samples could not be considered as certified reference standards, the median of the HIV-1 DNA levels obtained at all the study labs was taken as a reference for the assessment of interlaboratory reproducibility, as proposed in a previous HIV-1 DNA quantification quality assurance study^28^.

To assess the ability to recognize the most representative subtypes, seven HIV-1 strains obtained by CFAR (codes ARP1089, ARP1112, 100595, ARP 169.6, ARP1121, 100215 and ARP1124, representing the A1, B, C, D, F, CRF01_AE and CRF02_AG variants, respectively) were used to infect the lymphoblastoid MOLT-4/CCR5 cell line expressing high levels of CCR5 receptor (code ARP5039; CFAR) and DNA was extracted at the coordinator lab. HIV-1 DNA was originally quantified in HIV-1 subtype extracts at the coordinating lab to ensure appropriateness of the material, however, similar to RBSs analysis, the median values derived from the study were used to estimate target underestimation or overestimation with the different subtypes.

### Statistical analysis

Data were reported as mean ± SD or median (IQR) copies per test or copies per 10^6^ cells, as appropriate for the distribution of data based on the Shapiro–Wilk test for normality. Comparisons between independent groups of data were done by the Student t-test or by Mann–Whitney U test. Analysis of paired data was done by the paired t test or by Wilcoxon signed rank sum test. Comparisons between frequencies were done by chi-square test. The trend between ordered independent variables and continuous dependent variables was analyzed by the Jonckheere-Terpstra test. Statistical analysis was performed with SPSS version 20.0.

## Results

### Assays used for total HIV-1 DNA quantification

The main features of the assays used at the 12 participating centers are shown in Table 2. Data sets provided are indicated by the lab number followed by _qPCR or _ddPCR, consistent with the method used. Four labs performed homebrew qPCR, 3 homebrew ddPCR, and 3 both methodologies. The remaining 2 labs tested one of two different commercial qPCR assays each, namely the Generic HIV DNA Cell kit (Biocentric) and the HIV-1 DNA Test PRO (Diatheva) (lab07_qPCR and lab11_qPCR, respectively).

**Table 2:** Main features of the different methods used at each lab participating to the external quality assurance total HIV-1 DNA quantification program.

Lab	Extraction method	Method to determine the total DNA concentration		Method to determine the HIV-1 DNA concentration		PCR master mix	Instrument	References
		Method	Target	Method	Target			
lab01_qPCR	Abbott *m*Sample Preparation System DNA Kit on Abbott *m*2000*sp* instrument; Abbott	qPCR	hTERT	qPCR	Integrase	Abbott RealTime HIV-1 Amplification Reagent Kit; Abbott	7500 Fast Dx RTPCR Instrument; Applied Biosystems	^28,29^
lab02_qPCR	QIAamp DNA Blood Mini Kit; Qiagen	qPCR	Albumin	qPCR	pol	PrecisionPLUS qPCR Master Mix; PrimerDesign	RotorGene Q; Qiagen	^30^
lab03_ddPCR	High pure PCR template preparation kit; Roche	ddPCR	Albumin	ddPCR	LTR	ddPCR™ Supermix for Probes (No dUTP); Biorad	QX200 Droplet Digital PCR System; BioRad	^31^
lab04_ddPCR	AllPrep DNA/RNA Mini Kit; Qiagen	ddPCR	rpp30	ddPCR	gag	ddPCR™ Supermix for Probes (No dUTP); Biorad	QX200 Droplet Digital PCR System; BioRad	^32^
lab05_qPCR	Qiacube, Qiamp DNA mini kit; Qiagen	Spectrophotometry		qPCR	pol	iTaqUniversal probes supermix; Biorad	ABI 7900; Applied Biosystem	^33^
lab06_qPCR	High pure PCR template preparation kit; Roche	qPCR	Beta Globin	qPCR	LTR	GoTaq probe qPCR master Mix; Promega	Eco Real-Time PCR system; Illumina	^34^
lab06_ddPCR	High pure PCR template preparation kit; Roche	ddPCR	Albumin	ddPCR	LTR	ddPCR™ Supermix for Probes (No dUTP); Biorad	QX200 Droplet Digital PCR System; BioRad	^31^
lab07_qPCR	QIAamp DNA Blood Mini Kit; Qiagen	Spectrophotometry		qPCR	LTR	Generic HIV DNA Cell kit; Biocentric	QuantStudio 5 Dx Real-Time PCR System; Thermo Fisher	^35–37^Commercial kit
lab08_qPCR	QIA Symphony DNA Blood Mini Kit; Qiagen	qPCR	hTERT	qPCR	LTR	GoTaq Probe qPCR Reaction Mix; Promega	LightCycler 2.0; Roche	^34^
lab08_ddPCR	QIA Symphony DNA Blood Mini Kit; Qiagen	ddPCR	Albumin	ddPCR	LTR	ddPCR™ Supermix for Probes (No dUTP); Biorad	QX200 Droplet Digital PCR System; BioRad	^31,38^
lab09_ddPCR	High pure PCR template preparation kit; Roche	ddPCR	Albumin	ddPCR	LTR	ddPCR™ Supermix for Probes (No dUTP); Biorad	QX200 Droplet Digital PCR System; BioRad	^38^
lab10_qPCR	QIA Symphony DNA Blood Mini Kit; Qiagen	qPCR	Beta Globin	qPCR	LTR	homemade	RotorGene Q; Qiagen	^35^
lab11_qPCR	QIAamp DNA Blood Mini Kit; Qiagen	qPCR	hTERT	qPCR	LTR	HIV-1 DNA Test PRO; Diatheva	ABI 7500; Applied Biosystem	^39^ Commercial Kit
lab12_qPCR	High pure viral nucleic acid kit; Roche	qPCR	Albumin	qPCR	LTR	PreMix ExTaq; Takara	LightCycler 96; Roche	^27^
lab12_ddPCR	High pure viral nucleic acid kit; Roche	ddPCR	Albumin	ddPCR	LTR	ddPCR™ Supermix for Probes (No dUTP); Biorad	QX200 Droplet Digital PCR System; BioRad	^27^

*hTERT* human Telomerase Reverse Transcriptase.

### Sensitivity and linear range on titrated standards

**Figure 1:**
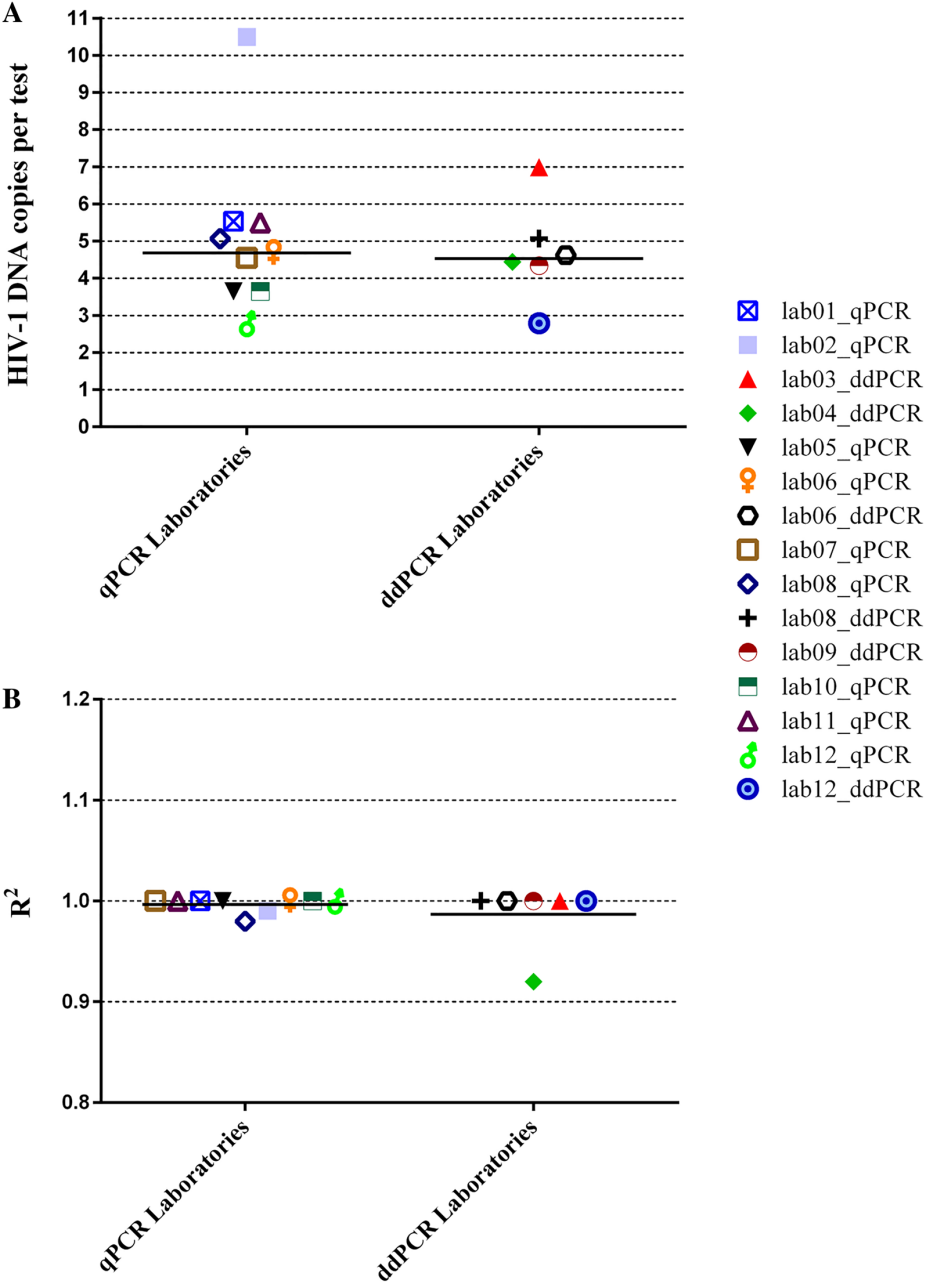
Distribution of the sensitivity, expressed as 95% hit rate (**A**), and linear range, expressed as R^2^ value on a log_10_ dilution series (**B**), of the different HIV-1 DNA quantification assays. Bars indicate median values. Graphic elaboration was realized using the GraphPad Prism software version 6.0 (https://www.graphpad.com/scientific-software/prism/).

The sensitivity, defined as the smallest amount of HIV-1 DNA detectable in the 95% of cases (95% hit rate) was calculated on 8 replicates of a twofold dilution series of the reference standard containing 1.75 to 112 nominal HIV-1 DNA copies in 50 ng of human DNA. Results were delivered as frequency of qualitative positive reactions for each standard dilution. Based on probit analysis, the median analytical sensitivity of the assays was 4.6 (3.7–5.5) HIV-1 DNA copies per test at the 95% hit rate, including one outlier result (10.5 HIV-1 DNA copies per test) and without statistically significant difference between qPCR and ddPCR (4.7 [3.7–5.5] *vs*. 4.5 [4.0–5.5] HIV-1 DNA copies per test respectively; *P* = 0.776) (Fig. 1A). When analyzing 200 to 1,000 ng of total DNA as in a typical clinical application, this translates into a limit of detection of around 150 to 30 HIV-1 DNA copies per 10^6^ cells, assuming as a reference that one million cells contain 6.66 µg of DNA and once confirmed that the same performance is maintained with clinical samples.

The linear dynamic range, as determined by testing a 5-point log_10_ dilution series of the standard (70,000 to 7 nominal copies per test), was expressed as the R^2^ of the linear regression. The median R^2^ value obtained was 1.000 (1.00–1.00), again without difference between qPCR and ddPCR (Fig. 1B). There was one outlier value (R^2^ = 0.92).

### Accuracy and precision on titrated standards

**Figure 2:**
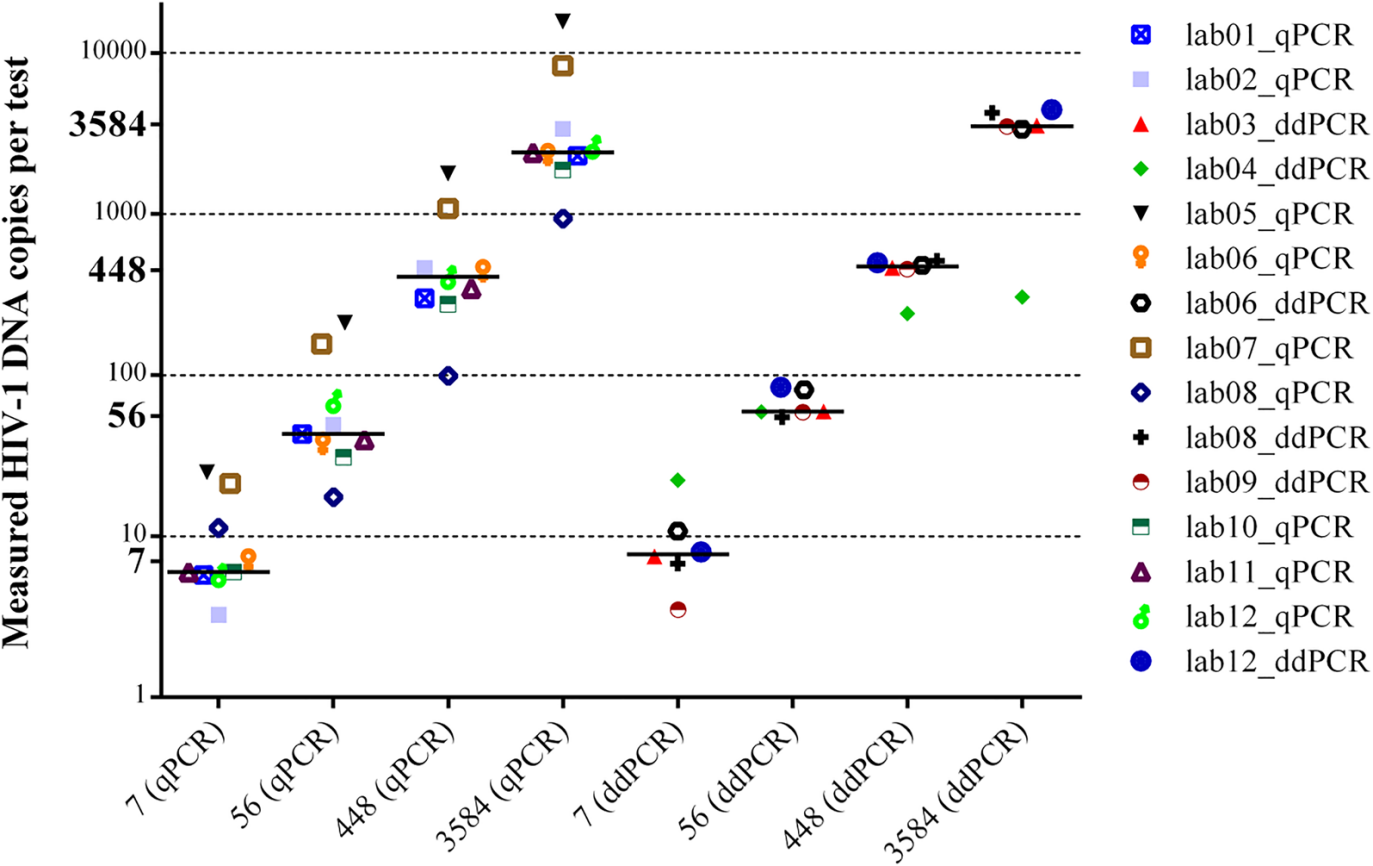
Expected and measured HIV-1 DNA copy values with the reference standard dilution series, stratified for qPCR and ddPCR. Bars indicate median values. Graphic elaboration was realized using the GraphPad Prism software version 6.0 (https://www.graphpad.com/scientific-software/prism/).

Accuracy was defined as the distance between measured and expected HIV-1 DNA copies and it was tested on 4 standard dilutions, each analyzed in quadruplicate. The median values obtained across labs were 3,370 (2,287–4,245), 445 (299–498), 59 (40–81) and 7 (6–11) HIV-1 DNA copies per test, for the 3,584, 448, 56 and 7-copy standards, respectively (Fig. 2). The ratio between nominal and measured values (fold difference) and the outlier values recorded by three labs are indicated in Supplementary Table 1. At individual sample level, fold-difference values were comparable across labs using ddPCR *vs.* qPCR (1.0 [0.7–1.2] *vs.* 0.7 [0.6–1.6], *P* = 0.456; 1.1 [0.9–1.1] *vs*. 0.9 [0.6–1.7], *P* = 0.388; 1.1 [1.0–1.5] *vs*. 0.8 [0.6–2.0], *P* = 0.224; 1.1 [0.8–1.9] *vs.* 0.9 [0.8–2.3], *P* = 0.607; for the 3,584, 448, 56 and 7-copy standards, respectively). When analyzing the whole fold-difference data set, there was a trend for ddPCR to yield higher values compared with qPCR (median fold-differences 1.1 [1.0–1.2] *vs.* 0.8 [0.6–2.0], *P* = 0.075). However, ddPCR values were significantly closer to the nominal copy numbers compared with qPCR (median absolute deviation 0.04 [0.02–0.17] vs. 0.19 [0.08–0.43] log, *P* = 0.002).

Precision was defined as the coefficient of variation (CV) of the HIV-1 DNA copies measured on 4 identical replicates of the standard at four different concentrations, both intra-run and inter-run (Supplementary Table 2). In the intra-run assessment, the median CV (considering both qPCR and ddPCR) was 5.5 [4.1–16.9], 9.9 [6.5–17.6], 20.9 [17.0–26.3] and 48.9 [33.4–71.5] for the 3,584, 448, 56 and 7-copy standards, respectively. CV values obtained by qPCR and ddPCR were comparable when considering the whole data set (18.8 [6.8–45.5] vs. 20.2 [5.7–32.0], respectively; *P* = 0.411) and for any individual reference standard (data not shown). In the inter-run assessment, testing the same dilution series in five independent runs, the median CV was 16.2 [7.9–21.0], 17.1 [8.9–22.5], 24.7 [18.0–28.5] and 46.0 [35.3–58.5] for the 3,584, 448, 56 and 7-copy standards, respectively. A higher CV was scored for qPCR *vs.* ddPCR when considering the whole inter-run data set (23.9 [18.1–37.3] *vs.* 17.9 [10.1–24.5], *P* = 0.02). This difference was more relevant at lower copy numbers (55.5 [38.5–71.7] *vs.* 37.4 [22.9–50.9], *P* = 0.088; 25.9 [22.7–31.6] *vs.* 17.3 [13.1–23.2], *P* = 0.018; 18.3 [12.4–22.9] *vs.* 9.8 [6.0–20.9], *P* = 0.181; 20.1 [9.3–21.5] *vs.* 11.8 [6.8–17.4], *P* = 0.328; at 7, 56, 448 and 3,584-copy numbers, respectively). Notably, both intra-run and inter-run CV values tended to increase with decreasing copy numbers (*P* = 0.042).

### Inter-laboratory variability and intra-laboratory precision on reconstructed blood samples

**Figure 3:**
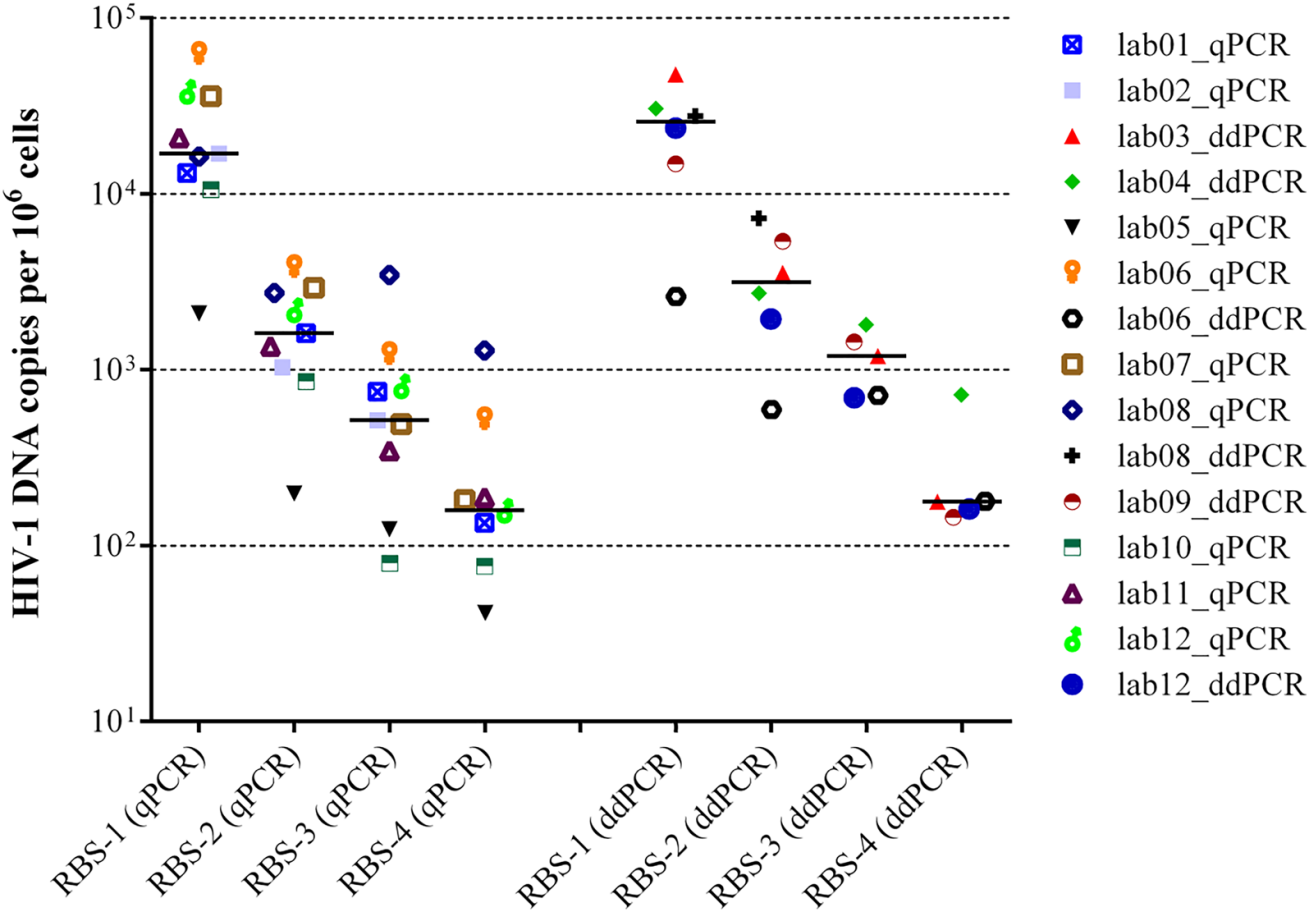
HIV-1 DNA (copies per 10^6^ cells) measured by the participating laboratories in the different reconstructed blood samples (RBS), stratified for qPCR and ddPCR. Bars indicate median values. Graphic elaboration was realized using the GraphPad Prism software version 6.0 (https://www.graphpad.com/scientific-software/prism/).

Each lab extracted the five RBSs (Table 1) in 3 independent runs using its own extraction protocol, then each DNA extract was quantified in duplicate in 3 independent experiments. Due to blood clots, lab08_ddPCR did not process RBS-3 and RBS-4. The median values obtained for RBS-1, RBS-2, RBS-3 and RBS-4 considering all the results delivered by the participating labs were 19,469 (11,020–31,805), 1,903 (946–3,030), 684 (184–1,285) and 145 (65–273) HIV-1 DNA copies per 10^6^ cells, respectively (Fig. 3). These values matched very closely those originally obtained at the coordinating lab (Table 1). When stratified by method, there was no statistically significant difference in ddPCR vs. qPCR values for any RBS (25,681 [11,755–34,906] vs. 16,977 [11,858–37,060], *P* = 0.776; 3,134 [1,605–5,843] vs. 1,610 [943–2,823], *P* = 0.224; 1,193 [703–1,622] vs. 516 [235–1,014], *P* = 0.190; 178 [154–449] vs. 159 [59–354], *P* = 0.606), although the median values were larger for ddPCR in all of the four samples.

When considering median values for each RBS as a reference, outlier values were detected for lab08_qPCR with RBS-3 and RBS-4, for lab06_qPCR with RBS-1 and RBS-4, for lab08_ddPCR with RBS-2 and for lab02_qPCR, lab04_ddPCR and lab05_qPCR with RBS-4. Thus, the lowest copy-number RBS (RBS-4) originated a larger number of outliers compared to the other RBSs (*P* = 0.074). Lab02_qPCR failed to detect HIV-1 DNA in RBS-4, this sample was excluded from the inter-laboratory variability analysis. The fold-difference between the measured and median values for each sample and lab are indicated in Supplementary Table 3. Overall, an absolute fold-difference > 2 was scored for one third of measurements, namely 4/15, 5/15, 5/14 and 4/13 for the four RBSs ordered by decreasing HIV-1 DNA titer, respectively, with no cases differing by more than 1 log with respect to the reference median value. When comparing fold-differences obtained by qPCR and ddPCR for all the individual RBSs, qPCR values were significantly closer to the median compared with ddPCR values (median 1.0 [0.6–1.6] *vs.* 1.4 [1.1–2.5], *P* = 0.039). This difference was prevalently driven by RBS-2 and RBS-3 (ratio between average ddPCR and qPCR values above twofold). Notably, the negative blood sample was scored as positive in 3 replicates (811.2 ± 615.3 HIV-1 copies per 10^6^ cells) by lab04_ddPCR, in 9 replicates (110.0 ± 73.1 HIV-1 copies per 10^6^ cells) by lab06_ddPCR and in 4 replicates (2.5 ± 1.0 HIV-1 copies per 10^6^ cells) by lab07_qPCR. Overall, the rate of false positive reactions was significantly higher for ddPCR *vs.* qPCR (12/108 [11.1%] *vs.* 4/162 [2.5%], *P* = 0.003).

Precision on the same RBS panel was determined by measuring the CV both within and across extraction runs (Supplementary Table 4). The intra-extraction precision was calculated as the median CV of three extraction runs while the inter-extraction precision was calculated as the median CV of all the 18 replicates. In the intra-extraction assessment, the median CV was 20.7 (12.0–29.7), 24.8 (20.0–45.1), 35.8 (24.6–65.0) and 51.2 (31.4–67.4) for RBS-1, RBS-2, RBS-3 and RBS-4, respectively. In the inter-extraction assessment, the median CV for the same series was 25.0 (14.3–59.7), 33.7 (25.6–48.7), 50.3 (37.4–84.1) and 64.9 (36.1–82.4). Similar to what found with plasmid standards, there was a significant increase in CV values with decreasing HIV-1 DNA copy numbers, both in the intra- and inter-extraction assessment (*P* = 0.042). The median CVs observed in the inter-extraction assessment were significantly higher than those observed in the intra-extraction assessment (*P* < 0.01). No significant differences in CVs obtained by qPCR or ddPCR were observed for either the whole RBS panel or any individual RBS.

### Ability to recognize different subtypes

**Figure 4:**
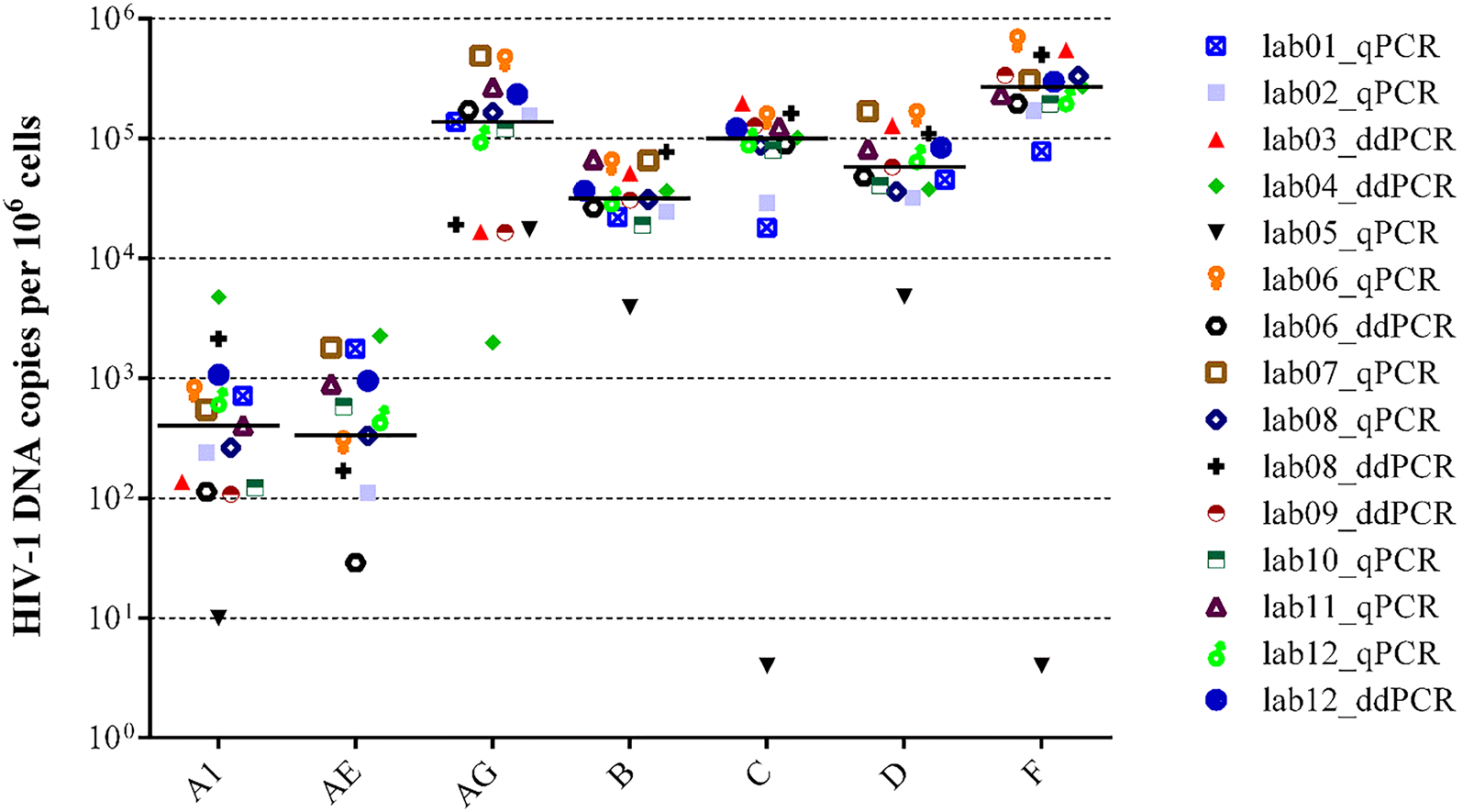
HIV-1 DNA (copies per 10^6^ cells) measured by the participating laboratories in the DNA extracts from different HIV-1 subtypes. Bars indicate median values. Graphic elaboration was realized using the GraphPad Prism software version 6.0 (https://www.graphpad.com/scientific-software/prism/).

To assess the ability to recognize and quantify the main HIV-1 subtypes, the coordinator lab sent to each lab seven tubes containing DNA extracted from a lymphoblastoid cell line infected with reference subtypes A1, CRF01_AE, CRF02_AG, B, C, D, F (Table 1). The median number of HIV-1 DNA copies per 10^6^ cells obtained at all labs was: 404 (123–404) for subtype A, 333 (29–948) for CRF01_AE, 137,040 (17,316–233,579) for CRF02_AG, 31,305 (24,450–31,305) for subtype B, 99,560 (67,704–131,654) for subtype C, 57,975 (37,700–109,194) for subtype D and 269,932 (194,573–337,652) for subtype F (Fig. 4). Measured HIV-1 DNA was > 1 log lower than the median in 6 cases: CRF02_AG by lab04_ddPCR; subtypes A1, C, D, F by lab05_qPCR; CRF01_AE by lab06_ddPCR. In addition, CRF01_AE was not detected at all by lab03_ddPCR, lab05_qPCR and lab09_ddPCR (Supplementary Table 5). By contrast, there was only one case where measured HIV-1 DNA was > 1 log higher than the median (lab04_ddPCR with subtype A1).

Alignment of available primer and probe sequences on the consensus of the different subtypes included in the panel revealed that, of the four labs underestimating or missing CRF01_AE, three used a forward primer affected by a 1-base deletion when hybridizing to the CRF01_AE and subtype F target sequence (Supplementary Fig. 1). Underestimation of CRF02_AG by lab04_qPCR coincided with the largest number of mismatches (6) affecting the forward primer among all the target subtypes considered, including one mismatch at position -3 bases with respect to the primer 3’ end. Finally, lab05_qPCR primers and probes perfectly matched the consensus B sequence but were affected by 4–9 mismatches when aligned to the other subtypes.

## Discussion

Despite conceptual limitations, total HIV-1 DNA is broadly considered as a measure of the viral reservoir and an attractive marker to monitor its changes following specific treatment strategies. Due to the lack of certified systems for in vitro diagnostic use, several homebrew assays have been developed, based on qPCR^27,29,40^ or ddPCR^31^. However, the heterogeneity of methods may affect interpretation of data generated across different labs due to different sensitivity, accuracy and precision. Only two previously published papers^28,40^ reported a multicenter quality control to evaluate the inter-laboratory reproducibility of total HIV-1 DNA quantification. In the French study^40^, 4 peripheral blood mononuclear cell (PBMC) samples were tested by 10 labs all using the commercially available Biocentric system. In the Italian study^28^, 7 labs tested 24 cellular samples carrying HIV-1 clade B and 40 HIV-1-negative PBMC samples spiked with different concentrations of plasmids containing the *gag* gene derived from different HIV-1 subtypes. All the labs participating to the latter study used hTERT as the housekeeping gene and one of two different primer sets to quantify HIV-1 DNA. The current study considerably expanded the scope of the quality control panel to assess the assays for their sensitivity, accuracy, linear range, precision and ability to recognize the different HIV-1 variants (Table 1). In addition, the variety of procedures used allowed an assessment of homebrew methods *vs.* commercial kits and of qPCR *vs.* ddPCR.

In total, 15 data sets were delivered by the 12 labs participating to the Italian HIV DNA Network. The overall performance with reference standards was good. Indeed, the linear range and sensitivity were excellent, with R^2^ = 1.000 in 12/15 data sets and the 95% hit rate of 4.6 copies of target which translates into a sensitivity threshold of 150 to 30 copies of HIV-1 DNA per million cells when using 200 to 1,000 ng of total DNA in the reaction, provided PCR is run under optimal conditions without any inhibition. Also, the accuracy and precision measured on the titrated standards were high, with the difference from the expected value within twofold in 46/60 cases (15 centers testing 4 samples) and the CV < 50% in 44/60 and 47/60 cases in the intra- and inter-run assessment, respectively. However, an overall lower performance was obtained with RBSs. While the nominal number of copies was not certified for the RBSs, the difference from the median value, derived from the values obtained by all labs, was within twofold only in 36/58 cases. The CV was < 50% in 42/58 and 35/58 cases in the intra- and inter-extraction assessment, respectively. Although there may remain differences from clinical blood samples, the RBSs were prepared from a single source of HIV-1-negative human blood spiked with HIV-1-positive cells from infected cell lines, thus mirroring blood from infected patients. RBSs were then frozen to reproduce the most common material used in the clinic. The lower precision with RBSs compared to ready-to-use plasmid standards likely derived from additional sources of variability including HIV-1 DNA extraction and measurement. Noteworthy, precision appeared to decrease with lower numbers of target copies, as often occurring with individuals under prolonged successful therapy^37^. In addition, repeated RBS analysis revealed lower precision in inter-extraction compared with intra-extraction runs. These drawbacks may advise for same-run replicate analysis of samples obtained at different time points from the same patient in the clinical setting.

Comparative analysis of the 9 qPCR *vs.* the 6 ddPCR data sets revealed few significant differences. With titrated standards, ddPCR results matched more closely than qPCR the expected target copy numbers; in addition, qPCR had larger inter-run CV values with respect to ddPCR, particularly at low-copy numbers. With RBSs, ddPCR values tended to distribute above the median of the whole dataset and also generated a significantly higher rate of false positive reactions compared with qPCR when analyzing the negative control. Higher precision with respect to qPCR is indeed one of the expected benefits of ddPCR, particularly at low copy numbers^41^. The distribution of ddPCR generated values with RBSs may reflect the qPCR underestimation bias detected with plasmid standards. Indeed, median values obtained for the whole dataset were influenced more by qPCR values (9 datasets) than by ddPCR (6 datasets). On the other hand, the trend for ddPCR to generate some false positive signals might have derived from an incorrect setting of the threshold, a well-known key issue with ddPCR complicating low-level detection abilities and requiring training by expert users or system manufacturers^42,43^. Overall, these specific caveats highlighted by our study suggest using replicate testing with qPCR and advise for inclusion of multiple negative controls and adjustments in the computation of background noise with ddPCR.

Of the 9 qPCR data sets, only 2 were generated by commercial, research-use-only kits. There were no relevant differences between homebrew and commercial qPCR results. However, the Biocentric system yielded the only few cases of false positive qPCR results with RBSs and also overestimated HIV-1 DNA copies in the titrated standards (2.3, 2.4, 2.8, 3.0-fold for the 3,584, 448, 56 and 7-copy standards, respectively) but not in the RBSs. Nevertheless, the availability of standardized and ready to use reagents from a commercial source remains valuable, particularly for labs with limited experience, and ensures large-scale validation as well as updates of primers and probes which should minimize false negative reactions.

Significant issues were scored with the HIV-1 subtype panel. Substantial underestimation of HIV-1 DNA occurred at several labs, with CRF01_AE most affected. The primary reason for underestimation or failure to detect a specific subtype is suboptimal primers and probes, possibly chosen from outdated literature or published studies focusing on specific subtypes. The ability of published primers and probes to recognize different HIV-1 subtypes should be verified on a regular basis by checking for conservation of the target regions in the updated reference databases such as GenBank and the curated Los Alamos National Laboratory repository. For example, underestimation or even lack of detection of CRF01_AE by four participants to this study may have been driven by the occurrence of one extra nucleotide in the region targeted by the forward primer. By contrast, lab08_ddPCR using the same primer/probe combination correctly quantified CRF01_AE, implying that other factors such as mismatch tolerant experimental conditions may have a role. However, it must be noted that CRF01_AE was also the lowest concentration DNA extract provided for subtype analysis which may have impacted accuracy per se. Similarly, lab04_qPCR likely underestimated CRF02_AG due to six mismatches in the forward primer. Finally, underestimation of multiple subtypes by lab05_qPCR apparently derived from choosing primers and probe targeting HIV-1 subtype B without adequate consideration for target conservation across different subtypes.

This comprehensive external quality assurance study documented good performance parameters for non-certified quantitative HIV-1 DNA assays, however significant caveats were documented, including subtype specific issues and false positive reactions. Since the study involved only labs with HIV-1 DNA quantification documented in peer-reviewed papers, the same performance may be not guaranteed in other labs running the assay outside of these inclusion criteria. However, the above shortcomings need to be addressed at any individual lab willing to provide HIV-1 DNA measurements either in research studies or in the clinical setting, irrespective from the experience in the field.

## Supplementary Information

Supplementary Information.
