# Supplementary material for: External quality assessment of HIV-1 DNA quantification assays used in the clinical setting in Italy
Source: Sci Rep. 2022 Feb 28;12:3291. doi: 10.1038/s41598-022-07196-2 (PMC8885833; doi:10.1038/s41598-022-07196-2)

SUPPLEMENTARY MATERIALS

**Supplementary Table 1.** Fold-difference between nominal and measured values obtained for the reference standard dilution series. Values in bold indicate outliers.

| Lab | Fold-difference at nominal HIV-1 DNA copies per test | | | |
| --- | --- | --- | --- | --- |
|  | 3584 copies per test | 448 copies per test | 56 copies per test | 7 copies per test |
| lab01_qPCR | 0.6 | 0.7 | 0.8 | 0.8 |
| lab02_qPCR | 0.9 | 1.0 | 0.9 | 0.5 |
| lab03_ddPCR | 1.0 | 1.0 | 1.1 | 1.1 |
| lab04_ddPCR | **0.1** | 0.5 | 1.1 | **3.2** |
| lab05_qPCR | **4.4** | **4.0** | **3.8** | **3.6** |
| lab06_ddPCR | 0.9 | 1.1 | 1.4 | 1.5 |
| lab06_qPCR | 0.6 | 1.0 | 0.7 | 1.0 |
| lab07_qPCR | **2.3** | **2.4** | **2.8** | **3.0** |
| lab08_ddPCR | 1.2 | 1.1 | 1.0 | 1.0 |
| lab08_qPCR | 0.3 | 0.2 | 0.3 | 1.6 |
| lab09_ddPCR | 1.0 | 1.0 | 1.1 | 0.5 |
| lab10_qPCR | 0.5 | 0.6 | 0.6 | 0.9 |
| lab11_qPCR | 0.7 | 0.8 | 0.7 | 0.9 |
| lab12_ddPCR | 1.2 | 1.1 | 1.5 | 1.1 |
| lab12_qPCR | 0.7 | 0.9 | 1.2 | 0.8 |
| Median (IQR)  qPCR | 0.7 (0.6-1.6) | 0.9 (0.6-1.7) | 0.8 (0.6-2.0) | 0.9 (0.8-2.3) |
| Median (IQR)  ddPCR | 1.0 (0.7-1.2) | 1.1 (0.9-1.1) | 1.1 (1.0-1.5) | 1.1 (0.8-1.9) |
| Median (IQR)  total | 0.9 (0.6-1.2) | 1.0 (0.7-1.1) | 1.1 (0.7-1.5) | 1.0 (0.8-1.6) |

**Supplementary Table 2.** Intra-run and inter-run assessment of precision with the reference standard dilution series. CV: coefficient of variation.

| Lab | Intra-run CV (nominal copy numbers) | | | | Inter-run CV (nominal copy numbers) | | | |
| --- | --- | --- | --- | --- | --- | --- | --- | --- |
|  | 3584 | 448 | 56 | 7 | 3584 | 448 | 56 | 7 |
| lab01_qPCR | 4.2 | 13.6 | 48.4 | 45.7 | 20.1 | 23.2 | 18.0 | 79.2 |
| lab02_qPCR | 33.9 | 29.2 | 72.3 | 145.1 | 21.0 | 30.7 | 51.9 | 89.4 |
| lab03_ddPCR | 5.8 | 5.5 | 5.7 | 56.0 | 12.1 | 8.8 | 14.2 | 19.9 |
| lab04_ddPCR | 23.7 | 62.0 | 21.3 | 28.0 | 16.2 | 10.8 | 25.8 | 46.0 |
| lab05_qPCR | 6.5 | 8.6 | 20.0 | 30.5 | 22.1 | 13.3 | 28.5 | 43.9 |
| lab06_ddPCR | 4.5 | 11.4 | 20.4 | 33.4 | 11.5 | 19.6 | 9.9 | 49.6 |
| lab06_qPCR | 31.9 | 73.9 | 33.4 | 44.9 | 17.8 | 17.1 | 20.7 | 58.5 |
| lab07_qPCR | 4.5 | 4.6 | 24.5 | 55.5 | 5.7 | 11.5 | 25.9 | 38.9 |
| lab08_ddPCR | 2.6 | 9.9 | 20.0 | 48.9 | 21.3 | 24.7 | 22.4 | 54.8 |
| lab08_qPCR | 16.9 | 17.6 | 15.1 | 15.2 | 7.4 | 8.9 | 24.7 | 55.4 |
| lab09_ddPCR | 4.1 | 6.5 | 26.3 | 85.7 | 3.5 | 2.6 | 20.3 | 23.9 |
| lab10_qPCR | 0.4 | 12.1 | 20.9 | 62.4 | 11.2 | 22.5 | 28.6 | 64.2 |
| lab11_qPCR | 5.7 | 6.5 | 17.0 | 81.6 | 24.7 | 18.5 | 24.9 | 38.0 |
| lab12_ddPCR | 1.3 | 7.0 | 23.2 | 43.3 | 7.9 | 7.1 | 14.2 | 28.7 |
| lab12_qPCR | 5.5 | 7.6 | 5.2 | 71.5 | 20.3 | 18.3 | 34.7 | 35.3 |
| Median (IQR)  qPCR | 5.7 (4.3-24.4) | 12.1 (7.0-23.4) | 20.9 (16.0-41.0) | 55.5 (38.1-71.5) | 20.1 (9.3-21.5) | 18.3 (12.4-22.8) | 25.9 (21.9-31.6) | 55.4 (38.5-50.9) |
| Median (IQR)  ddPCR | 4.3 (2.3-10.3) | 8.4 (6.2-24.0) | 20.8 (16.4-24.0) | 46.1 (32.0-63.4) | 11.8 (6.8-17.4) | 9.8 (6.0-20.9) | 17.3 (13.1-23.2) | 37.4 (22.9-50.9) |
| Median (IQR)  total | 5.5 (4.1-16.9) | 9.9 (6.5-17.6) | 20.9 (17.0-26.3) | 49.5 (33.4-71.5) | 15.9 (7.9-21.0) | 15.3 (8.9-22.5) | 24.7 (18.0-28.5) | 46.0 (35.3-56.8) |

**Supplementary Table 3.** Fold-differences between measured and median HIV-1 DNA copies in the whole data set. The median values for the reconstructed blood samples RBS-1, RBS-2, RBS-3 and RBS-4 were 19,469 (11,020-31,805), 1,903 (946-3,030), 684 (184-1,285) and 145 (65-273)DNA copies per 10^6^ cells, respectively. Values in bold indicate outliers.

| Lab | RBS-1 | RBS-2 | RBS-3 | RBS-4 |
| --- | --- | --- | --- | --- |
| lab01_qPCR | 0.7 | 0.8 | 1.1 | 0.9 |
| lab02_qPCR | 0.9 | 0.5 | 0.8 | ND* |
| lab03_ddPCR | 2.5 | 1.9 | 1.7 | 1.2 |
| lab04_ddPCR | 1.6 | 1.4 | 2.6 | **5.0** |
| lab05_qPCR | 0.1 | 0.1 | 0.2 | **0.3** |
| lab06_ddPCR | 0.1 | 0.3 | 1.0 | 1.2 |
| lab06_qPCR | 3.2 | 2.0 | 1.8 | **3.6** |
| lab07_qPCR | 1.8 | 1.5 | 0.7 | 1.3 |
| lab08_ddPCR | 1.4 | 3.8 | NP** | NP** |
| lab08_qPCR | 0.8 | 1.4 | **5.1** | **8.9** |
| lab09_ddPCR | 0.8 | **2.8** | 2.1 | 1.0 |
| lab10_qPCR | 0.5 | 0.4 | 0.1 | 0.5 |
| lab11_qPCR | 1.1 | 0.7 | 0.5 | 1.3 |
| lab12_ddPCR | 1.2 | 1.0 | 1.0 | 1.1 |
| lab12_qPCR | 2.0 | 1.2 | 1.2 | 1.1 |
| Median (IQR)  qPCR | 1.0 (0.6-2.2) | 0.8 (0.5-1.6) | 0.9 (0.4-1.7) | 1.3 (0.7-3.3) |
| Median (IQR)  ddPCR | 1.5 (0.7-2.1) | 1.7 (0.9-3.2) | 2.0 (1.2-2.7) | 1.3 (1.2-3.4) |
| Median (IQR)  total | 1.2 (0.7-2.1) | 1.2 (0.6-2.0) | 1.2 (0.7-2.1) | 1.3 (1.0-2.7) |

*HIV-1 DNA not detected.

**Not processed due to blood clotting.

**Supplementary Table 4.** Assay precision with reconstructed blood samples (RBS). The coefficient of variation (CV) was determined both intra-extraction (3 subsequent runs, in duplicate for each extraction) and inter-extraction (3 subsequent extractions, 18 replicates).

| lab | Intra-extraction CV | | | | Inter-extraction CV | | | |
| --- | --- | --- | --- | --- | --- | --- | --- | --- |
|  | RBS-1 | RBS-2 | RBS-3 | RBS-4 | RBS-1 | RBS-2 | RBS-3 | RBS-4 |
| lab01_qPCR | 6.2 | 14.1 | 46.0 | 66.0 | 14.9 | 31.1 | 55.7 | 63.7 |
| lab02_qPCR | 95.7 | 106.9 | 92.1 | ND* | 90.0 | 112.5 | 84.3 | ND* |
| lab03_ddPCR | 14.1 | 48.9 | 56.0 | 66.0 | 14.3 | 47.8 | 56.3 | 70.6 |
| lab04_ddPCR | 14.5 | 25.3 | 18.5 | 51.2 | 21.8 | 26.8 | 39.0 | 82.8 |
| lab05_qPCR | 6.3 | 10.8 | 16.3 | 16.5 | 22.3 | 10.7 | 49.4 | 64.9 |
| lab06_ddPCR | 63.9 | 45.1 | 165.0 | 77.5 | 60.2 | 48.7 | 188.8 | 147.2 |
| lab06_qPCR | 29.7 | 33.7 | 98.5 | 80.7 | 59.7 | 47.4 | 90.9 | 93.0 |
| lab07_qPCR | 7.5 | 10.6 | 24.1 | 60.5 | 10.7 | 55.1 | 38.1 | 81.9 |
| lab08_ddPCR | 23.2 | 47.4 | NP** | NP** | 24.0 | 62.6 | NP** | NP** |
| lab08_qPCR | 34.6 | 20.0 | 24.8 | 36.0 | 64.4 | 21.6 | 84.1 | 36.5 |
| lab09_ddPCR | 12.0 | 24.7 | 34.0 | 68.8 | 13.6 | 30.5 | 35.3 | 71.7 |
| lab10_qPCR | 26.7 | 24.3 | 51.3 | 44.8 | 31.6 | 25.6 | 60.5 | 49.6 |
| lab11_qPCR | 14.1 | 26.8 | 36.7 | 30.9 | 33.7 | 33.7 | 40.3 | 32.1 |
| lab12_ddPCR | 20.7 | 23.5 | 35.0 | 31.9 | 25.0 | 29.4 | 35.5 | 35.7 |
| lab12_qPCR | 29.5 | 24.8 | 31.2 | 30.1 | 32.2 | 24.1 | 30.1 | 30.9 |
| Median (IQR)  qPCR | 26.7 (6.9-32.1) | 24.3 (12.4-30.3) | 36.7 (24.4-71.7) | 40.4 (30.3-64.6 | 32.2 (17.2-62.1) | 33.7 (22.9-51.2) | 51.3 (39.2-84.2) | 51.3 (39.2-84.2) |
| Median (IQR)  ddPCR | 17.6 (13.6-33.3) | 35.2 (24.4-47.8) | 35.0 (26.2-110.5) | 66.0 (41.5-73.1 | 22.9 (14.1-33.8 | 39.1 (28.7-52.2) | 39.0 (35.4-122.5) | 39.0 (35.4-122.5) |
| Median (IQR)  total | 20.7 (12.0-29.7) | 24.8 (20.0-45.1) | 35.8 (24.6-65.0) | 51.2 (31.4-67.4) | 25.0 (14.3-59.7) | 33.7 (25.6-48.7) | 50.3 (37.4-84.1) | 64.9 (36.1-82.4) |

*HIV-1 DNA not detected.

**Not processed due to blood clotting.

**Supplementary Table 5.** Fold-differences between HIV-1 DNA copies measured and median value obtained for the whole data set with viral isolates carrying different HIV-1 subtypes.

| Lab | Subtype | | | | | | |
| --- | --- | --- | --- | --- | --- | --- | --- |
|  | A1 | B | C | CRF01_AE | CRF02_AG | D | F |
| lab01_qPCR | 1.75 | 0.70 | 0.18 | 5.29 | 1.00 | 0.78 | 0.29 |
| lab02_qPCR | 0.60 | 0.78 | 0.29 | 0.33 | 1.13 | 0.55 | 0.63 |
| lab03_ddPCR | 0.34 | 1.63 | 1.98 | ND* | 0.12 | 2.21 | 2.01 |
| lab04_ddPCR | 11.79 | 1.16 | 1.02 | 6.79 | 0.01 | 0.65 | 1.00 |
| lab05_qPCR | 0.02 | 0.12 | ND* | ND* | 0.13 | 0.08 | ND* |
| lab06_ddPCR | 0.28 | 0.85 | 0.89 | 0.09 | 1.26 | 0.82 | 0.72 |
| lab06_qPCR | 1.88 | 1.93 | 1.46 | 0.86 | 3.18 | 2.62 | 2.36 |
| lab07_qPCR | 1.36 | 2.09 | NP** | 5.34 | 3.57 | 2.89 | 1.13 |
| lab08_ddPCR | 5.26 | 2.45 | 1.62 | 0.51 | 0.14 | 1.88 | 1.85 |
| lab08_qPCR | 0.65 | 0.99 | 0.88 | 1.00 | 1.20 | 0.62 | 1.22 |
| lab09_ddPCR | 0.27 | 0.98 | 1.28 | ND* | 0.12 | 1.00 | 1.25 |
| lab10_qPCR | 0.30 | 0.61 | 0.81 | 1.74 | 0.88 | 0.70 | 0.72 |
| lab11_qPCR | 1.00 | 2.15 | 1.25 | 2.68 | 1.96 | 1.41 | 0.86 |
| lab12_ddPCR | 2.64 | 1.17 | 1.22 | 2.85 | 1.70 | 1.45 | 1.10 |
| lab12_qPCR | 1.64 | 1.00 | 0.98 | 1.41 | 0.75 | 1.22 | 0.80 |
| Mean±SD | 1.99±3.02 | 1.24±0.66 | 1.07±0.49 | 2.41±2.25 | 1.14±1.09 | 1.26±0.82 | 1.14±0.58 |

*HIV-1 DNA not detected.

**Not Processed due to a lab accident.

Supplementary Figure 1. Alignment of primer and probe sequences used at the individual labs on the consensus sequence of the different subtypes included in the study. The HXB2 subtype B sequence appears in the first line as a reference. To accommodate the different primers and probes, the alignments show the LTR (a), a region of the *gag* gene (b), and two different regions of the *pol* gene (c and d). Dots indicate homology with the reference HXB2, dashes indicate a missing nucleotide. The end of the LTR is marked to show that the reverse primers match across the borders between the LTR and the pre-gag region. Graphic elaboration was realized using the BioEdit Sequence Alignment Editor software version 7.1.3.0 (https://bioedit.software.informer.com/7.1/).


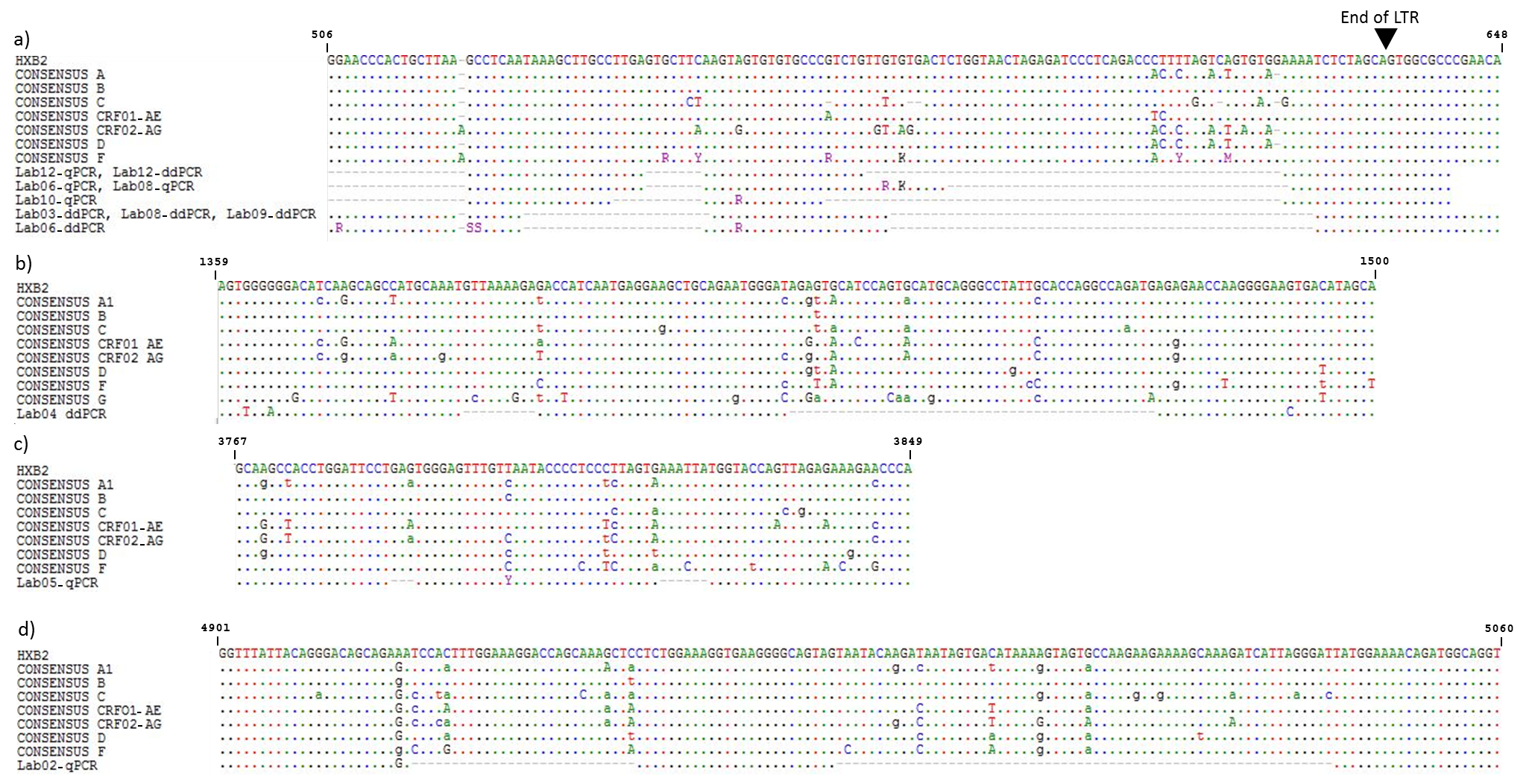

Supplement: Supplementary file 1 — Supplementary Information. [file 41598_2022_7196_MOESM1_ESM.docx]
